# Esophageal cancer mortality trends in the United States: a comprehensive longitudinal study (1999–2023) using CDC WONDER data

**DOI:** 10.3389/fpubh.2025.1696850

**Published:** 2025-10-29

**Authors:** Xuefei Yang, Yinyan Shao, Junhua Guo, Ting Huang, Heran Zhou

**Affiliations:** ^1^Department of Oncology, Hangzhou TCM Hospital Affiliated to Zhejiang Chinese Medical University, Hangzhou, Zhejiang, China; ^2^Department of General Medicine, Hangzhou TCM Hospital Affiliated to Zhejiang Chinese Medical University, Hangzhou, Zhejiang, China

**Keywords:** CDC WONDER, esophageal cancer, age-adjusted mortality rate, mortality trends, epidemiology

## Abstract

**Background:**

Esophageal cancer continues to pose a significant public health challenge worldwide. However, the extent to which advancements in treatment have reduced mortality at the population level remains unclear. This study examines trends in esophageal cancer mortality in the United States from 1999 to 2023, focusing on variations based on sex, ethnicity, urbanization level, census region, and age group.

**Methods:**

Mortality data were obtained from the Centers for Disease Control and Prevention’s Wide-Ranging Online Data for Epidemiologic Research (CDC WONDER) database using ICD-10 codes (C15.0–C15.9) to identify esophageal cancer-related deaths. The analysis included individuals aged 25 years and older. Temporal trends in age-adjusted mortality rates (AAMR) were analyzed using the Joinpoint Regression Program. Data were stratified by census region, metropolitan/non-metropolitan residence, and state. Annual percentage change (APC) and average annual percentage change (AAPC) were calculated along with their 95% confidence intervals (CI).

**Results:**

Between 1999 and 2023, a total of 357,606 deaths from esophageal cancer were recorded. A significant decline in the mortality rate was observed over this period, with the overall AAMR decreasing from 6.74 to 5.61 per 100,000, corresponding to an AAPC of −0.81* (*p-value < 0.05). Decline in the mortality rate was evident across nearly all ethnic groups, with the exception of the non-Hispanic (NH) white group. The most significant reduction was observed among non-Hispanic Black individuals (AAPC: −4.07*). Significant sex-based disparities persisted throughout the study period, with men consistently experiencing higher mortality rates than women. Geographically, mortality trends diverged: metropolitan areas experienced a significant decline (AAPC: −1.09*), whereas non-metropolitan areas experienced a significant increase (AAPC: 0.48*). Pronounced regional disparities were also noted, with the western and northeastern regions demonstrating the most substantial improvements. Age-specific analyses revealed a significant reduction in the mortality rate across the majority of age groups; however, among adults aged 85 years and older, the mortality rates remained stable.

**Conclusion:**

Despite an overall decline in the mortality rate of esophageal cancer, significant disparities persist across geographic, urban–rural, and age subgroups. These findings highlight the need for targeted public health interventions to address ongoing inequalities, particularly among NH white individuals, those living in non-metropolitan areas, and older adult.

## 1 Introduction

Esophageal cancer remains a major global public health challenge and a leading cause of cancer-related mortality. In the United States (1), it ranks among the top 10 deadliest cancers, with approximately 20,000 new cases and over 16,000 deaths annually (2). The major histological subtypes, adenocarcinoma and squamous cell carcinoma, exhibit distinct etiological and demographic profiles, contributing to the heterogeneity in incidence and survival rates (3). Despite advances in treatment, prognosis remains poor, with a 5-year survival rate below 20%, underscoring the urgent need for improved prevention and early detection strategies (4).

Social and demographic factors, including geography, ethnicity, and socioeconomic status, significantly influence the incidence and mortality rate of esophageal cancer. Previous studies, such as those utilizing the Surveillance, Epidemiology, and End Results (SEER) database, have reported persistently high mortality rates among non-Hispanic (NH) Black men and rural residents (5). Meanwhile, the incidence of esophageal adenocarcinoma has been increasing in Western populations, a trend associated with obesity and gastroesophageal reflux disease (6). However, many existing studies have limited geographical or demographic scope, resulting in a significant gap in understanding recent national trends. Therefore, a comprehensive analysis encompassing multiple demographic and geographical dimensions is required to elucidate these disparities and guide public health strategies.

To address this gap, the study utilizes the extensive Centers for Disease Control and Prevention’s Wide-Ranging Online Data for Epidemiologic Research (CDC WONDER) database to examine trends in esophageal cancer mortality in the United States from 1999 to 2023 (7). We analyzed age-adjusted mortality rates based on sex, ethnicity, urbanization level, census region, and age group. By identifying high-risk populations and evolving trends, this study aims to inform targeted public health interventions and reduce the national burden of esophageal cancer.

## 2 Materials and methods

### 2.1 Study setting and population

Data on esophageal cancer mortality between 1999 and 2023 were obtained from the National Vital Statistics System (NVSS) using the CDC WONDER platform (accessed 1 September 2025). This comprehensive database includes information on over 99% of US decedents across all 50 states and the District of Columbia. Mortality data, current through 31 December 2023, were extracted for underlying causes of death classified under ICD-10 codes (C15.0–C15.9). The analysis was restricted to adults aged 25 years and older, as esophageal cancer is exceedingly rare in younger populations, and data in CDC WONDER were insufficient for reliable analysis. We retrieved data related to demographic variables including age, sex, race, ethnicity, and cause of death. Age-adjusted mortality rates were calculated using the year 2000 US standard population and have been presented per 100,000 person-years. Data suppression was applied in accordance with CDC WONDER protocols for unreliable estimates (counts <10). This study followed the Strengthening the Reporting of Observational Studies in Epidemiology (STROBE) guidelines (8), and the datasets are publicly available at https://wonder.cdc.gov/.

### 2.2 Data abstraction

The dataset comprises information on population size, year, and the place of death, as well as demographic characteristics such as age, sex, and ethnicity. Ethnicity were classified according to the bridged-race estimates. Individuals of Hispanic or Latino origin were included as a separate category encompassing all ethnic backgrounds. NH groups were defined as mutually exclusive and included the following: white, Black or African American, American Indian or Alaska Native (AI/AN), and Asian or Pacific Islander (API). The “NH other” category, as provided by CDC WONDER, consists of individuals not otherwise classified into the preceding NH groups. Records with unknown ethnicity were excluded from ethnicity-stratified analyses. Geographic regions were defined according to the U.S. Census Bureau classifications: Northeast, Midwest, South, and West. Urban–rural status was assessed using the 2013 National Center for Health Statistics (NCHS) Urban–Rural Classification Scheme. To maintain consistency across the study period (1999–2023) and mitigate potential misclassification due to the changes in county designations over time, a fixed version of the 2013 scheme was employed. Counties were further grouped into three categories: large central and fringe metropolitan (population ≥1 million), medium and small metropolitan (population 50,000–999,999), and non-metropolitan (population < 50,000). Mortality data were obtained from death certificates and were consistent with prior studies utilizing the CDC WONDER database. Population estimates for rate calculations followed U.S. Census Bureau definitions of age, ethnicity, and geographic region (9).

### 2.3 Statistical analysis

This study included 357,606 decedents from 1999 to 2023. We calculated both crude and age-adjusted mortality rates (AAMRs) for esophageal cancer. AAMRs were standardized based on the year 2000 U.S. standard population to enable comparisons across demographic subgroups, including ethnicity, geographic region, and metropolitan/non-metropolitan status, while accounting for differences in age structure (10). For analyses stratified by age group, only crude mortality rates were reported. This approach was necessitated because the CDC WONDER platform does not support the calculation of age-adjusted rates within specific age brackets due to methodological constraints of applying a standard population to limited age ranges. Although crude rates do not adjust for finer variations in age structure within these broad categories, they are considered an appropriate and standard metric for presenting mortality trends within predefined age groups.

Temporal trends in mortality rates were analyzed using the Joinpoint Regression Program (Version 5.4.0.0; National Cancer Institute, USA). Analyses were configured, with a maximum of five joinpoints permitted. The model assumed uncorrelated errors, and a permutation test was applied with 4,499 permutations and an overall alpha level of 0.05 to determine the optimal number of joinpoints. The annual percent change (APC) and average annual percent change (AAPC) were reported along with their 95% confidence intervals (CI). A *p*-value ≤ 0.05 was considered statistically significant. Given the exploratory and descriptive nature of this epidemiological study, which aimed to identify potential trends across key demographic strata, consistent with common practice in analogous surveillance studies utilizing joinpoint regression, we did not adjust for multiple comparisons. The primary objective was to characterize trends within each prespecified subgroup, rather than testing a single overarching hypothesis. This analytical approach is consistent with prior studies that used CDC WONDER data (11).

### 2.4 Sensitivity analyses

We conducted sensitivity analyses to assess the robustness of our primary findings: (1) To evaluate modeling assumptions, we compared joinpoint trends with simple log-linear models over key intervals (1999–2005 and 2005–2023). (2) To test the stability of recent trends, we re-estimated the 2005–2023 model after excluding the pandemic years (2020–2021) and separately analyzed the 2019–2023 period for female individuals and by urbanization level (data through 2020). (3) To address the use of crude rates in age-stratified analysis, we calculated directly standardized rates (DSRs) for broad age bands (45–64, 65–74, 75–84, and ≥85 years) and applied a Kitagawa decomposition method (2005–2023) to quantify the contribution of population aging.

### 2.5 Ethics approval

This study did not require institutional review board approval, as the CDC WONDER database contains anonymized, publicly available data.

## 3 Results

### 3.1 Overall

**Figure 1:**
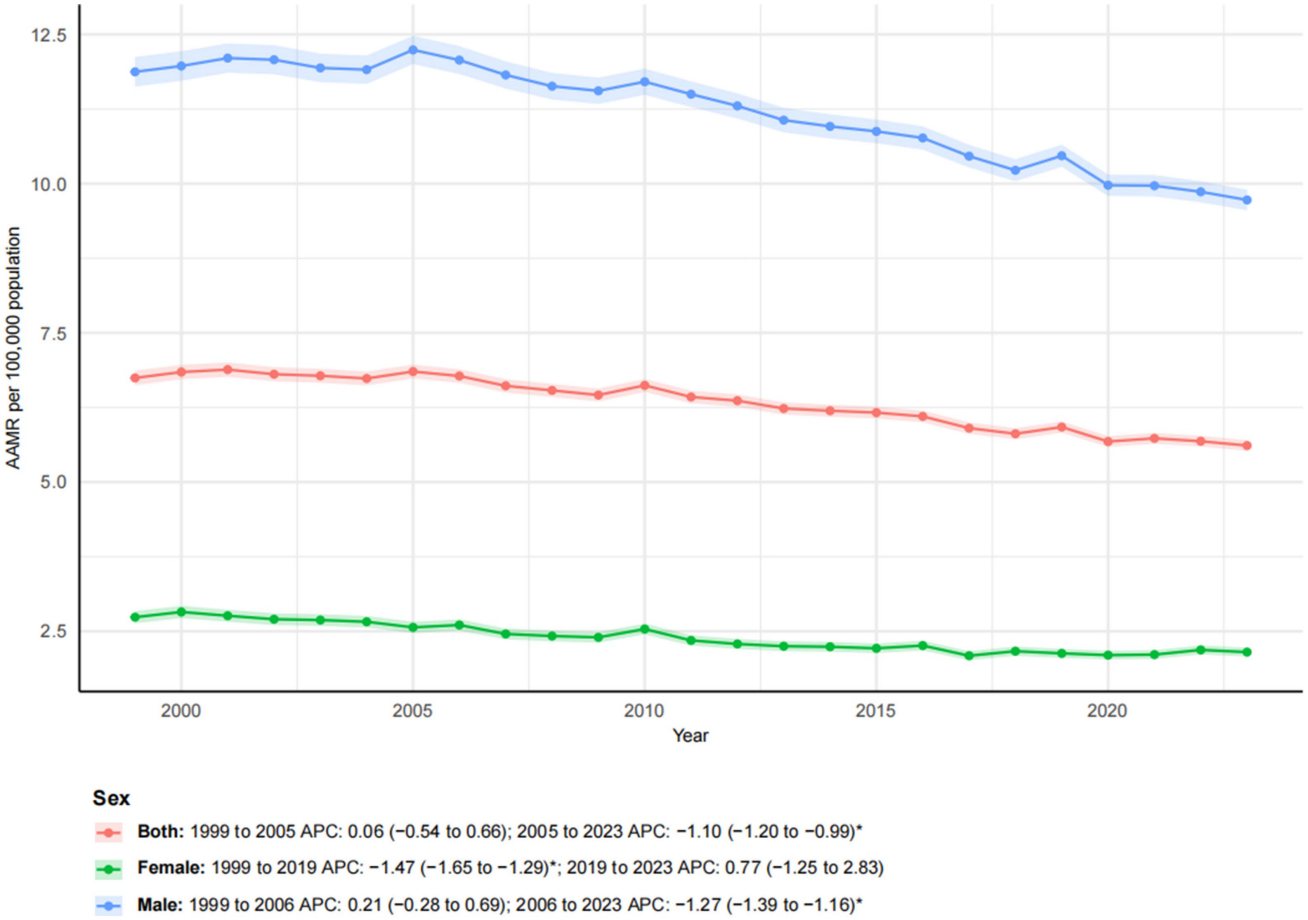
Trends in esophageal cancer-related age-adjusted mortality rates stratified by sex, 1999–2023. Asterisks indicate statistical significance at *p*-value < 0.05 (two-sided).

From 1999 to 2023, a total of 357,606 esophageal cancer-related deaths were recorded in the United States. The overall age-adjusted mortality rate (AAMR), standardized to the 2000 U. S. population, significantly declined from 6.74 (95% CI, 6.62–6.87) to 5.61 per 100,000, with an AAPC of −0.81* (95% CI, −0.97 to −0.65; *p-value < 0.05). In joinpoint analysis, there was no significant change during 1999–2005 (APC 0.06; 95% CI, −0.54 to 0.66), followed by a significant decline during 2005–2023 (APC -1.10*; 95% CI, −1.20 to −0.99; Figure 1). Concurrently, total deaths increased while AAMR decreased, indicating that population growth and aging contributed to the observed pattern, which has been explicitly illustrated in the age pyramid (Supplementary Figure S1) and through Kitagawa decomposition analysis of rate change (Supplementary Figure S2).

### 3.2 Sex

From 1999 to 2023, a pronounced sex-related disparity in esophageal cancer mortality was observed. Male individuals consistently had higher mortality rates than female individuals. The number of deaths among male individualss increased by 40.09% compared to a more moderate increase of 17.28% among female individuals. The AAMR declined for both sexes, from 11.87 to 9.73 per 100,000 in male individuals and from 2.74 to 2.15 per 100,000 in female individuals. Joinpoint regression revealed distinct temporal trends for each sex (Figure 1). For male individuals, mortality showed a non-significant increase from 1999 to 2006 (APC: 0.21), followed by a significant decline from 2006 to 2023 (APC: −1.27*; 95% CI: −1.39 to −1.16). In contrast, female mortality exhibited a significant decreasing trend from 1999 to 2019 (APC: −1.47*; 95% CI: −1.65 to −1.29), which was followed by a non-significant increase from 2019 to 2023 (APC: 0.77). These patterns highlighted persistent sex-based disparities and indicated that temporal trends were not uniform throughout the study period.

### 3.3 Ethnicity

**Figure 2:**
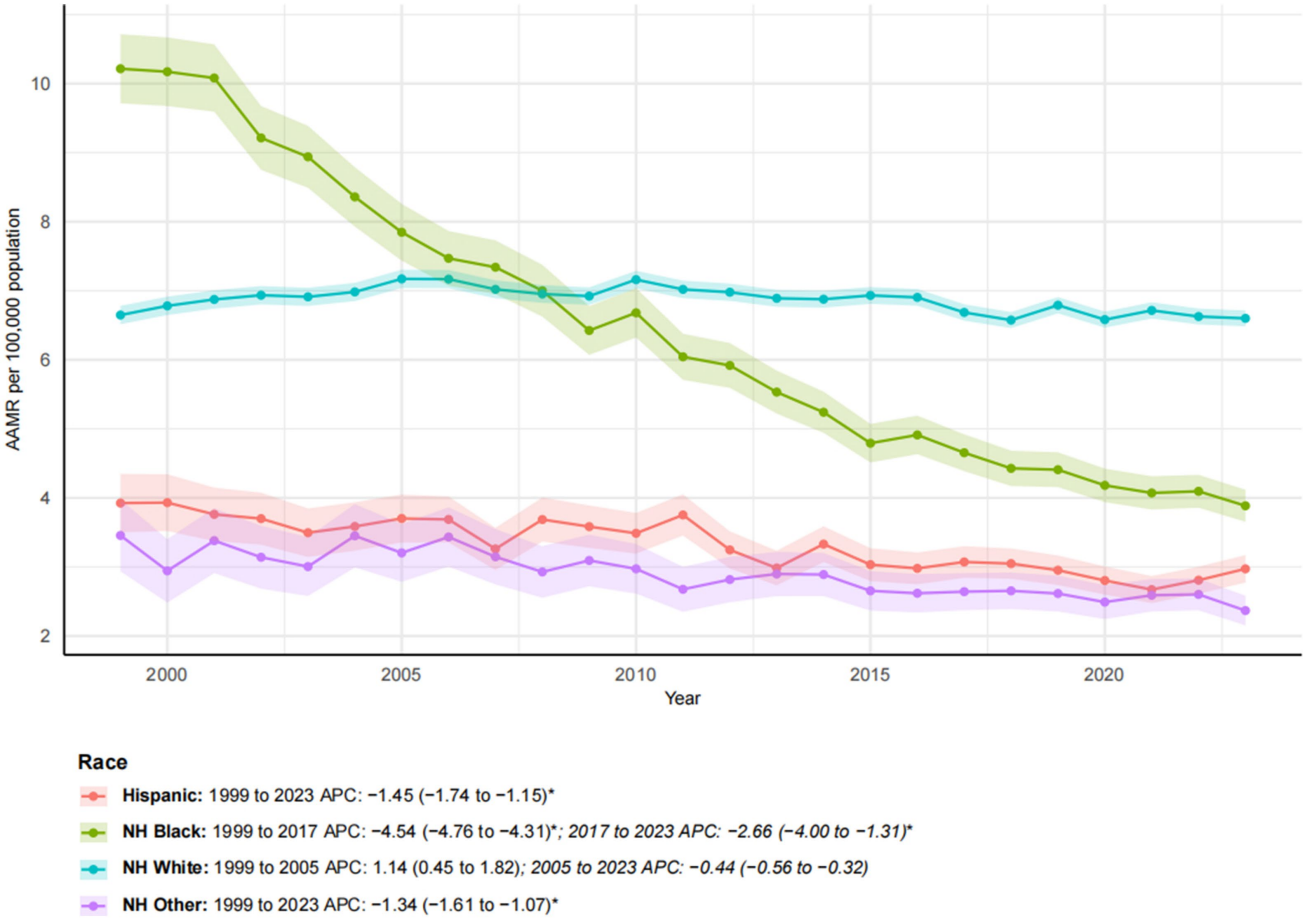
Trends in esophageal cancer-related age-adjusted mortality rates stratified by race, 1999–2023. Asterisks indicate statistical significance at *p*-value < 0.05 (two-sided).

From 1999 to 2023, notable racial and ethnic disparities in esophageal cancer mortality trends were observed (Figure 2). NH Black individuals experienced the most substantial improvement, with a 30.04% reduction in mortality. The AAMR decreased significantly from 10.21 to 3.89 per 100,000. The AAPC was −4.07* (95% CI: −4.42 to −3.72), reflecting a decline from 1999 to 2017 (APC: −4.54*; 95% CI: −4.76 to −4.31), followed by a slower yet significant decline from 2017 to 2023 (APC: −2.66*; 95% CI: −4.00 to −1.31). Similarly, the AAMR for Hispanic and NH other groups also exhibited significant downward trends (APC of −1.45* and −1.34*, respectively), despite substantial increases in the number of deaths (153.13 and 155.85%, respectively), reflecting both population growth and improvement in mortality rates. In contrast, the NH white population experienced a 38.90% increase in mortality, while the AAMR remained relatively stable, decreasing only slightly from 6.65 to 6.60 per 100,000, with a non-significant AAPC of −0.05. This trend exhibited a biphasic pattern: a non-significant increase from 1999 to 2005 (APC: 1.14), followed by a slight, non-significant decline from 2005 to 2023 (APC: −0.44).

### 3.4 Metropolitan/non-metropolitan

**Figure 3:**
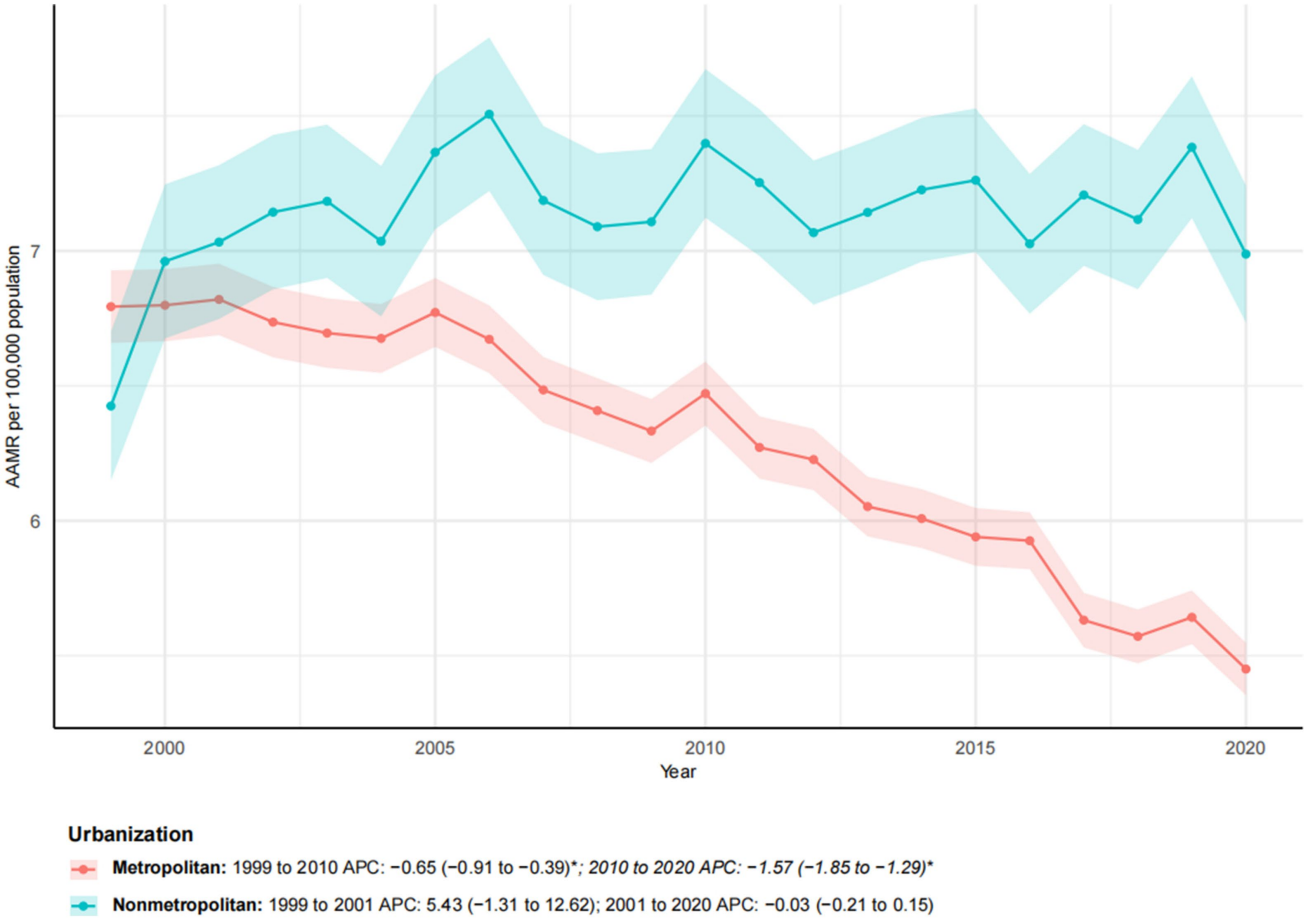
Trends in esophageal cancer-related age-adjusted mortality rates stratified by urbanization level, 1999–2023. Asterisks indicate statistical significance at *p*-value < 0.05 (two-sided).

Between 1999 and 2023, a significant difference was observed in esophageal cancer mortality trends among metropolitan and non-metropolitan areas (Figure 3). While the number of deaths in metropolitan areas increased by 30.22%, the AAMR decreased from 6.79 to 5.45 per 100,000, with a notable AAPC of −1.09* (95% CI: −1.26 to −0.91). In contrast, non-metropolitan areas experienced a more pronounced increase in mortality (55.55%), with the AAMR rising from 6.43 to 6.99 per 100,000. The overall AAPC for non-metropolitan areas was 0.48, indicating the absence of a significant downward trend. Segmental analysis revealed that metropolitan areas experienced a moderate decline from 1999 to 2010 (APC: −0.65*; 95% CI: −0.91 to −0.39), followed by a significantly accelerated decline from 2010 to 2020 (APC: −1.57*; 95% CI: −1.85 to −1.29). Conversely, non-metropolitan areas exhibited a sharp increase from 1999 to 2001 (APC: 5.43), followed by stagnation from 2001 to 2020 (APC: −0.03). These findings underscore growing metropolitan–non-metropolitan disparities, with improving outcomes in metropolitan areas contrasting with worsening mortality in non-metropolitan areas.

### 3.5 Census regions

**Figure 4:**
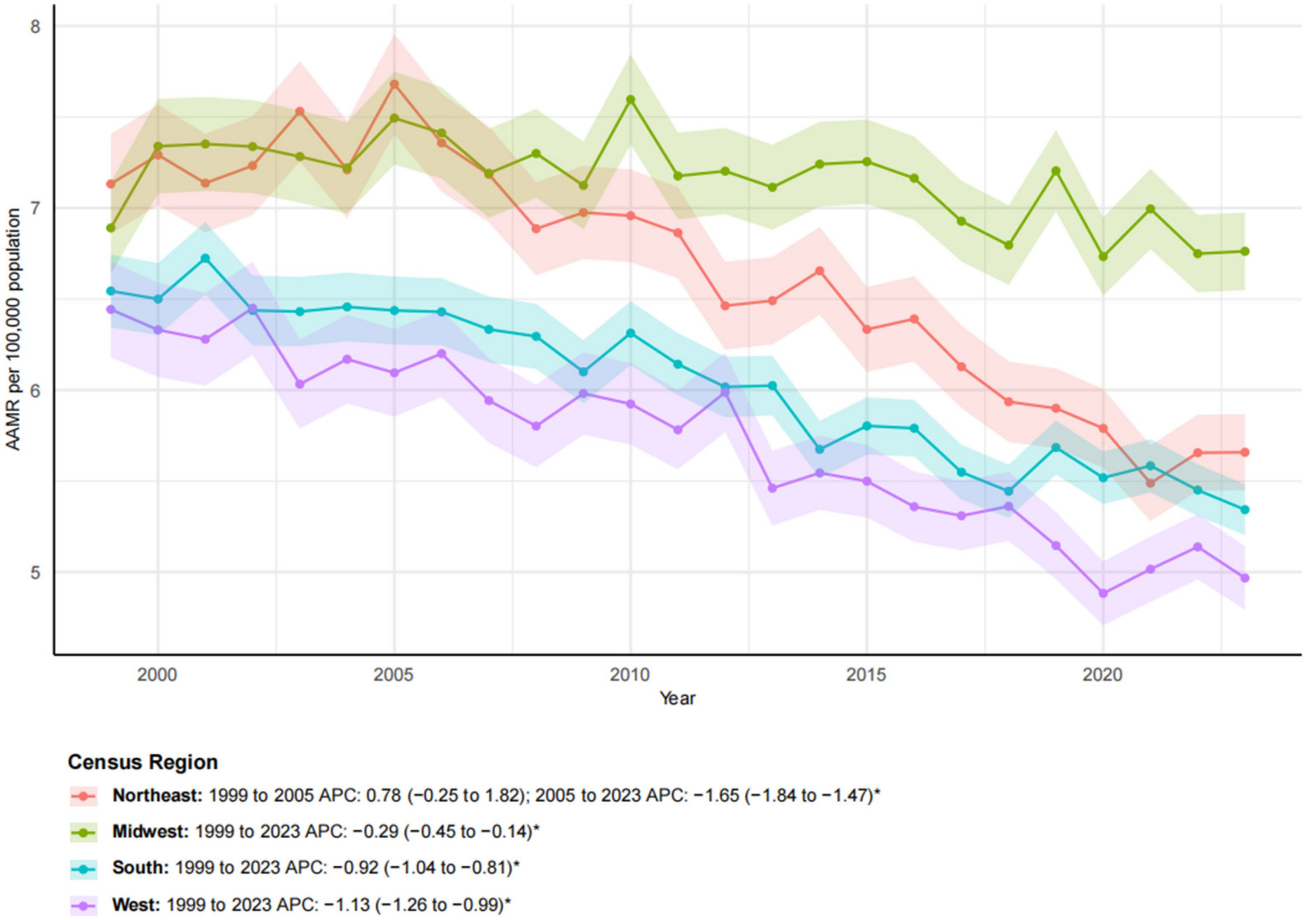
Trends in esophageal cancer-related age-adjusted mortality rates stratified by census region, 1999–2023. Asterisks indicate statistical significance at *p*-value < 0.05 (two-sided).

**Figure 5:**
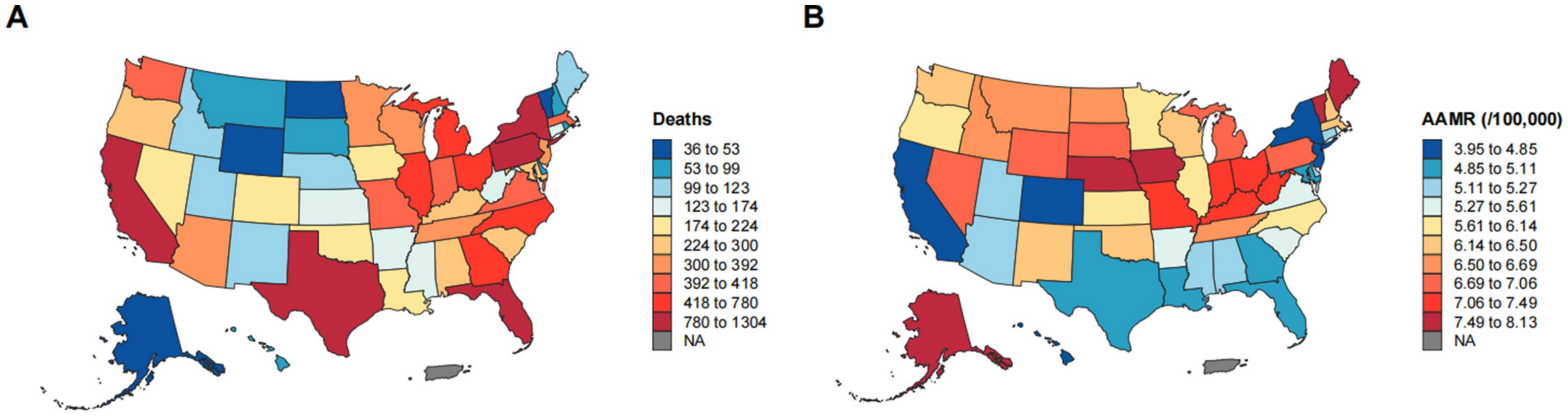
Geographic disparities in esophageal cancer mortality. **(A)** Geographic distribution of the number of deaths from esophageal cancer. **(B)** Geographic distribution of AAMR per 100,000 population for esophageal cancer.

From 1999 to 2023, significant geographic disparities in esophageal cancer mortality rates were observed across U. S. Census regions (Figures 4, 5). The Northeast region had the lowest increase in mortality (12.40%) and exhibited a biphasic trend: a non-significant rise from 1999 to 2005 (APC: 0.78), followed by a significant decline from 2005 to 2023 (APC: −1.65*; 95% CI: −1.81 to −1.47). Consequently, the AAMR decreased from 7.13 to 5.66 per 100,000, with an AAPC of −1.05* (95% CI: −1.33 to −0.78). The Western region demonstrated the most substantial improvement, with an AAPC of −1.13* (95% CI: −1.26 to −0.99). Despite a 39.65% increase in mortality, the AAMR fell from 6.44 to 4.97 per 100,000. The Southern region also exhibited a significant downward trend (AAPC: −0.92*; 95% CI: −1.04 to −0.81), with the AAMR declining from 6.54 to 5.34 per 100,000. In contrast, the Midwest region experienced a substantial increase in mortality (40.80%), yet the AAMR exhibited the lowest decrease (from 6.89 to 6.76 per 100,000) and the slowest downward trend (AAPC: −0.29*; 95% CI: −0.45 to −0.14). These patterns highlight significant regional differences, with the West and Northeast achieving a more pronounced reduction in esophageal cancer mortality, while progress in the Midwest and South has been more limited (Table 1).

**Table 1:** Trends in esophageal cancer mortality in the United States in 1999 and 2023, stratified by sex, census region, race, urbanization level, and age group.

Characteristic	Deaths			AAMR		AAPC (95% CI)
	1999	2023	Percent change (%)	1999 (95% CI)	2023 (95% CI)	
Overall	11917	16050	34.68	6.74 (6.62 to 6.87)	5.61 (5.52 to 5.70)	−0.81 (−0.97 to −0.65)*
Sex						
Female	2824	3312	17.28	2.74 (2.63 to 2.84)	2.15 (2.08 to 2.23)	−1.10 (−1.44 to −0.76)*
Male	9093	12738	40.09	11.87 (11.63 to 12.12)	9.73 (9.55 to 9.90)	−0.84 (−0.99 to −0.69)*
Census region						
Northeast	2620	2945	12.40	7.13 (6.86 to 7.41)	5.66 (5.45 to 5.87)	−1.05 (−1.33 to −0.78)*
Midwest	2875	4048	40.80	6.89 (6.64 to 7.14)	6.76 (6.55 to 6.98)	−0.29 (−0.45 to −0.14)*
South	4122	5845	41.80	6.54 (6.34 to 6.74)	5.34 (5.20 to 5.48)	−0.92 (−1.04 to −0.81)*
West	2300	3212	39.65	6.44 (6.18 to 6.71)	4.97 (4.79 to 5.14)	−1.13 (−1.26 to −0.99)*
Race						
Hispanic	367	929	153.13	3.92 (3.50 to 4.35)	2.97 (2.77 to 3.17)	−1.45 (−1.74 to −1.15)*
NH Black	1628	1139	−30.04	10.21 (9.71 to 10.72)	3.89 (3.65 to 4.12)	−4.07 (−4.42 to −3.72)*
NH White	9703	13477	38.90	6.65 (6.52 to 6.78)	6.60 (6.49 to 6.71)	−0.05 (−0.23 to 0.13)
NH Other	188	481	155.85	3.46 (2.94 to 3.98)	2.37 (2.15 to 2.58)	−1.34 (−1.61 to −1.07)*
Urbanization						
Metropolitan^#^	9818	12785	30.22	6.79 (6.66 to 6.93)	5.45 (5.35 to 5.55)^#^	−1.09 (−1.26 to −0.91)*
Nonmetropolitan^#^	2099	3265	55.55	6.43 (6.15 to 6.70)	6.99 (6.73 to 7.24)^#^	0.48 (−0.13 to 1.08)
Age						
25–34 years	37	47	27.03	0.09 (0.09 to 0.09)	0.10 (0.10 to 0.10)	0.33 (−0.62 to 1.29)
35–44 years	293	217	−25.94	0.65 (0.65 to 0.65)	0.49 (0.49 to 0.49)	−1.36 (−1.75 to −0.97)*
45–54 years	1262	986	−21.87	3.45 (3.45 to 3.45)	2.43 (2.43 to 2.43)	−1.68 (−2.01 to −1.35)*
55–64 years	2531	3300	30.38	10.64 (10.64 to 10.64)	7.88 (7.88 to 7.88)	−1.25 (−1.51 to −0.99)*
65–74 years	3694	5517	49.35	20.06 (20.06 to 20.06)	15.91 (15.91 to 15.91)	−1.05 (−1.14 to −0.97)*
75–84 years	2986	4279	43.30	24.43 (24.43 to 24.43)	23.30 (23.30 to 23.30)	−0.41 (−0.73 to −0.10)*
85+ years	1114	1704	52.96	26.82 (26.82 to 26.82)	27.51 (27.51 to 27.51)	−0.07 (−0.30 to 0.16)

*AAPC is statistically significant (*p* < 0.05). ^#^AAMR for urbanization in 2023 uses 2020 classification criteria. AAMR for age groups are crude mortality rates; AAPC was calculated based on crude rates. AAMR, age-adjusted mortality rate; CI, confidence interval; AAPC, average annual percent change.

### 3.6 Age groups

**Figure 6:**
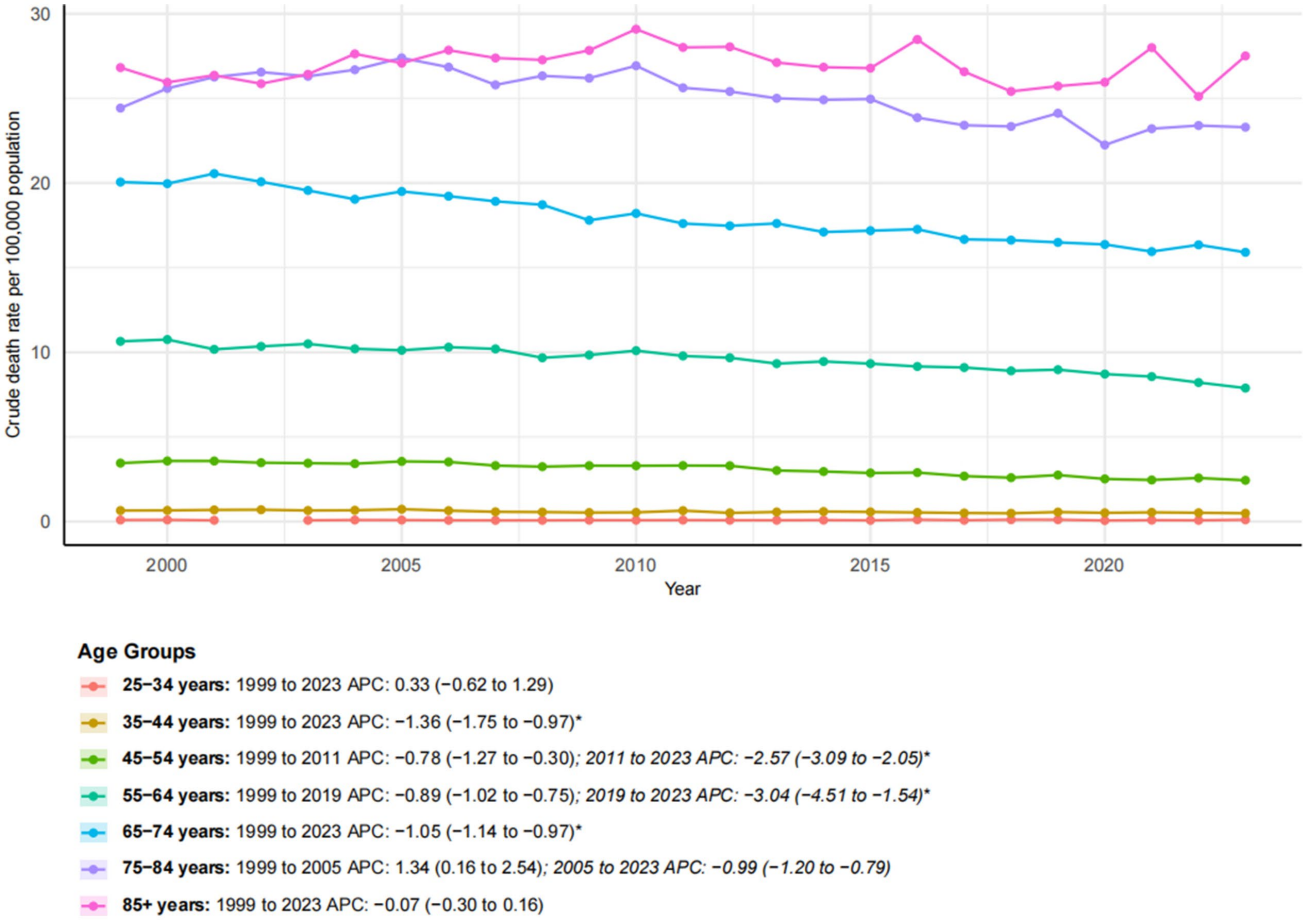
Trends in esophageal cancer-related crude mortality rates stratified by age groups, 1999–2023. Asterisks indicate statistical significance at *p*-value < 0.05 (two-sided).

Age-specific trends in esophageal cancer mortality revealed distinct patterns from 1999 to 2023 (Figure 6). Younger adults (ages 25–44) experienced very low mortality rates, with the 35–44 age group experiencing a significant decline (AAPC: −1.36*; 95% CI: −1.75 to −0.97). Middle-aged groups (ages 45–64) showed considerable improvements, particularly after 2011 for the 45–54 age group (APC: −2.57*; 95% CI: −3.09 to −2.05) and after 2019 for the 55–64 age group (APC: −3.04*; 95% CI: −4.51 to −1.54), indicating accelerated recent progress. Older age groups exhibited more varied trends. Individuals aged 65–74 and 75–84 experienced a significant decline in mortality (AAPC: −1.05* and −0.41*, respectively), although their absolute rates remained high. In contrast, the oldest group (ages 85 and older) showed no significant change over the study period (AAPC: −0.07) and maintained the highest mortality rate in 2023 (27.51 per 100,000). These findings underscore the progress made in reducing esophageal cancer mortality among most adult age groups and the persistent challenges in the oldest population.

### 3.7 Sensitivity analyses

Sensitivity analyses supported the robustness of our primary findings (Supplementary Table S1; Supplementary Figures S3–S6). Exclusion of the pandemic years (2020–2021) did not substantially affect the overall declining mortality trends for either males (2005–2023 APC: -1.26, 95% CI: −1.35 to −1.17 vs. -1.23, −1.33 to −1.14) or females (APC: −1.14, −1.38 to −0.89 vs. -1.11, −1.39 to −0.83). However, among females, a subsequent non-significant increase was noted from 2019 to 2023 (APC: 0.61, −0.34 to 1.57).

Analysis of urban–rural disparities through 2020 revealed a persistent decline in metropolitan areas (2005–2020 APC: −1.36, −1.50 to −1.22), in contrast to a stagnation in non-metropolitan regions (APC: −0.17, −0.37 to 0.04). To further validate age-stratified results, we calculated directly standardized rates (DSRs) for broad age categories (45–64, 65–74, 75–84, and ≥85 years), which consistently mirrored trends observed in crude mortality rates (Supplementary Tables S2, S3). A Kitagawa decomposition analysis (2005–2023) further quantified the substantial contribution of population aging to overall changes in crude mortality (Supplementary Figure S2). Finally, shifts in the adult population structure between 1999 and 2023, marked by pronounced growth and aging, have been depicted in the Supplementary Figure S1.

## 4 Discussion

Our comprehensive analysis of U. S. esophageal cancer mortality from 1999 to 2023 revealed a complex and evolving epidemiological landscape. Although the overall AAMR declined significantly, this trend conceals substantial disparities across sex, race, urbanization level, census region, and age group. These findings emphasize the heterogeneous burden of esophageal cancer and identify populations that have not benefited equally from broader national improvements.

The observed overall decline in esophageal cancer mortality is consistent with previous national reports and likely reflects advancements in treatment modalities, including surgical techniques and radiation therapy, and the introduction of advanced therapies, such as immunotherapy (12). Additionally, changing patterns in known risk factors, particularly the decline in smoking and alcohol consumption, which are major contributors to esophageal squamous cell carcinoma, may have contributed to this trend (13). Crucially, the robustness of this long-term decline was supported by our sensitivity analyses, which confirmed that the trend remained stable both after excluding the pandemic years and in analyses using prespecified piecewise fits. Similarly, the apparent uptick in female mortality during 2019–2023 was small and not statistically significant across these analyses, suggesting it is more likely a random fluctuation than evidence of a sustained reversal. However, the stabilization or reversal of declining trends in certain subgroups is concerning and suggests that not all populations are benefiting equally from medical advancements.

A key finding of this study is the significant racial and ethnic disparity in mortality trends. The dramatic decline among NH Black individuals, while encouraging, must be interpreted in the context of their persistently elevated risk factors and historical disparities in access to timely diagnosis and high-quality care (14). The relatively high mortality rate among NH white individuals at the end of the study period, especially compared to the marked declines in other groups, is a critical finding. We hypothesize that this pattern may be partly influenced by the rising incidence of esophageal adenocarcinoma, which is strongly associated with obesity and gastroesophageal reflux disease and is more prevalent in the NH white population (15), as supported by external incidence studies. The contrasting trends suggest a shifting epidemiological paradigm in which the unequal distribution of emerging risk factors is reshaping traditional disparities.

It is also instructive to contextualize these U. S. trends within the global burden of esophageal cancer. In high-incidence regions such as Eastern Asia and Eastern Africa, esophageal squamous cell carcinoma remains the dominant histological subtype, largely attributable to tobacco use, alcohol consumption, and dietary factors such as hot beverage intake and nitrosamine-rich foods. While the U.S. has experienced a gradual decline in overall mortality, these regions continue to face persistently high incidence and mortality rates, underscoring the influence of regional risk factor profiles and healthcare infrastructure. Comparisons with such populations may further highlight the distinct drivers of esophageal cancer in the U.S., particularly the growing prominence of adenocarcinoma in the Western population.

The stark and growing disparity between metropolitan and non-metropolitan areas is a major concern. Our sensitivity analyses reinforced that the decline in mortality was consistently concentrated in metropolitan areas, whereas non-metropolitan trajectories remained essentially flat. The rising mortality in non-metropolitan areas likely stems from multiple factors, including limited access to specialized care, lower rates of early detection, and a higher prevalence of risk factors such as smoking and obesity (16, 17). This finding aligns with broader trends of deteriorating health outcomes in rural America, underscoring the urgent need for targeted delivery models for early detection and treatment and other interventions such as the improvement of healthcare infrastructure and access in underserved regions.

Geographic variation further illustrates the uneven landscape of esophageal cancer mortality. The more pronounced improvements in the West and Northeast may be linked to higher population density, a greater concentration of academic medical centers and specialists, and early adoption of advanced screening and treatment protocols compared to the Midwest and South. Additionally, state-level policies regarding healthcare access, tobacco control, and cancer prevention initiatives contribute to these regional disparities.

The age-specific trends offer a nuanced perspective on progress across the life course. The significant decline in mortality among middle-aged adults (45–64 years) is particularly promising, as it represents a substantial gain in potential years of life saved. This can be attributed to the effectiveness of current treatments and a growing emphasis on earlier detection in symptomatic patients within this age range. In contrast, the stability of mortality in the oldest age group (85 years and older) presents a complex challenge. While this may reflect a higher burden of comorbidities that limit treatment options or a focus on palliative rather than curative care, it also highlights a potential gap in evidence-based management of the older adult (18).

This study is subject to several limitations inherent to its data sources and methodology: Potential coding inaccuracies and temporal variations in cause-of-death attribution within death certificate data, including transitions related to ICD-10 updates, and the absence of detailed clinical and socioeconomic information in the CDC WONDER database, such as cancer stage, histologic subtype, treatment modalities, and socioeconomic status, which limits a more nuanced interpretation of the observed disparities (19). Furthermore, small-number stabilization techniques and certain sensitivity analyses for racial subgroups could not be applied due to constraints in the database interface. Despite these limitations, the study’s strengths, including its national scope, extended temporal coverage, and comprehensive demographic examination, provide a robust overview of the U.S. esophageal cancer mortality landscape.

## 5 Conclusion

In conclusion, this study demonstrates that the overall decline in esophageal cancer mortality in the U.S. from 1999 to 2023 represents a promising yet incomplete achievement. The significant disparities we identified indicate an uneven distribution of progress. Increasing mortality rates in non-metropolitan areas and persistently high mortality among NH white and older adult populations highlight new challenges in the fight against esophageal cancer. Future efforts must complement broad national strategies with targeted and equitable interventions. They should focus on improving access to specialized care in non-metropolitan regions, promoting early detection strategies tailored to high-risk demographics, and advancing research on the management of esophageal cancer in older adult.

## Data Availability

The original contributions presented in the study are included in the article/Supplementary material, further inquiries can be directed to the corresponding author.

## Supplementary material

SUPPLEMENTARY FIGURE S1 Population age distribution of the United States in 1999 and 2023. Shown are the percentage shares by 5–year age groups for each year, normalized so that percentages sum to 100% within year. The figure highlights the shift toward older age groups over time.

SUPPLEMENTARY FIGURE S2 Kitagawa decomposition of crude rate change, 2005–2023 (ages ≥25 years). Contributions (per 100,000) of population composition (age structure) versus within-age mortality changes to the net change in crude mortality rate between 2005 and 2023. Positive bars indicate an increase in the overall crude rate; negative bars indicate a decrease.

SUPPLEMENTARY FIGURE S3 Esophageal cancer AAMR by sex, 1999–2023.Age-adjusted mortality rates (per 100,000) for males and females, standardized to the 2000 U.S. standard population (5–year age groups). Vertical axis shows AAMR; horizontal axis shows calendar year. AAMR, age-adjusted mortality rate.

SUPPLEMENTARY FIGURE S4 Female AAMR sensitivity to pandemic years. Comparison of female AAMR trends using all years versus excluding 2020–2021. Rates are standardized to the 2000 U.S. standard population (5–year age groups). AAMR, age-adjusted mortality rate.

SUPPLEMENTARY FIGURE S5 AAMR by urbanization, 1999–2020. Age-adjusted mortality rates for metropolitan versus nonmetropolitan areas, using the fixed 2013 NCHS Urban Rural Classification (county assignments held constant). Rates are standardized to the 2000 U.S. standard population (5–year age groups). NCHS, National Center for Health Statistics.

SUPPLEMENTARY FIGURE S6 Piecewise segments for sexes (1999–2005 vs 2005–2023). Sex-specific AAMR trends fit with simple log-linear models for the prespecified intervals 1999–2005 and 2005–2023. Rates standardized to the 2000 U.S. standard population (5–year age groups).

SUPPLEMENTARY TABLE S1 Sensitivity APCs (95% CIs) across segments and strata.

SUPPLEMENTARY TABLE S2 Age-specific death counts, population estimates, and crude mortality rates per 100,000, 1999–2023.

SUPPLEMENTARY TABLE S3 Directly standardized mortality rates (DSR) per 100,000 with 95% confidence intervals for broad age bands, 1999–2023.

## References

[1] Lander S Lander E Gibson MK. Esophageal Cancer: overview, risk factors, and reasons for the rise. Curr Gastroenterol Rep. (2023) 25:275–9. doi: 10.1007/s11894-023-00899-0, PMID: 37812328

[2] Sung H Ferlay J Siegel RL Laversanne M Soerjomataram I Jemal A. Global Cancer statistics 2020: GLOBOCAN estimates of incidence and mortality worldwide for 36 cancers in 185 countries. CA Cancer J Clin. (2021) 71:209–49. doi: 10.3322/caac.21660, PMID: 33538338

[3] Morgan E Soerjomataram I Rumgay H Coleman HG Thrift AP Vignat J. The global landscape of esophageal squamous cell carcinoma and esophageal adenocarcinoma incidence and mortality in 2020 and projections to 2040: new estimates from GLOBOCAN 2020. Gastroenterology. (2022) 163:649–58.e2. doi: 10.1053/j.gastro.2022.05.054, PMID: 35671803

[4] Yang H Wang F Hallemeier CL Lerut T Fu J. Oesophageal cancer. Lancet. (2024) 404:1991–2005. doi: 10.1016/S0140-6736(24)02226-8, PMID: 39550174

[5] Islami F Ward EM Sung H Cronin KA Tangka FKL Sherman RL. Annual report to the nation on the status of Cancer, part 1: National Cancer Statistics. J Natl Cancer Inst. (2021) 113:1648–69. doi: 10.1093/jnci/djab131, PMID: 34240195 PMC8634503

[6] Maret-Ouda J Markar SR Lagergren J. Gastroesophageal reflux disease: a review. JAMA. (2020) 324:2536–47. doi: 10.1001/jama.2020.21360, PMID: 33351048

[7] Minhas AMK Sperling LS Al-Kindi S Abramov D. Underlying and contributing causes of mortality from CDC WONDER-insights for researchers. Am Heart J Plus. (2025) 50:100499. doi: 10.1016/j.ahjo.2025.100499, PMID: 39895921 PMC11782113

[8] Cuschieri S. The STROBE guidelines. Saudi J Anaesth. (2019) 13:S31–4. doi: 10.4103/sja.SJA_543_18, PMID: 30930717 PMC6398292

[9] Ingram DD Franco SJ. NCHS urban-rural classification scheme for counties. Vital Health Stat 2. (2012) 154:1–65. 22783637

[10] Anderson RN Rosenberg HM. Age standardization of death rates: implementation of the year 2000 standard. Natl Vital Stat Rep. (1998) 47:1–20. 9796247

[11] Rahman HAU Fahim MAA Salman A Alim Ur Rahman H Ahmed Ali Fahim M Kumar S. Investigating sex, race, and geographic disparities in bronchus and lung cancer mortality in the United States: a comprehensive longitudinal study (1999-2020) utilizing CDC WONDER data. Ann Med Surg. (2024) 86:5361–9. doi: 10.1097/MS9.0000000000002387, PMID: 39238989 PMC11374286

[12] Harada K Rogers JE Iwatsuki M Yamashita K Baba H Ajani JA. Recent advances in treating oesophageal cancer. F1000Res. (2020) 9:F1000. doi: 10.12688/f1000research.22926.1 PMC7531047 33042518

[13] Huang FL Yu SJ. Esophageal cancer: risk factors, genetic association, and treatment. Asian J Surg. (2018) 41:210–5. doi: 10.1016/j.asjsur.2016.10.005, PMID: 27986415

[14] Lu CL Lang HC Luo JC Liu CC Lin HC Chang FY. Increasing trend of the incidence of esophageal squamous cell carcinoma, but not adenocarcinoma, in Taiwan. Cancer Causes Control. (2010) 21:269–74. doi: 10.1007/s10552-009-9458-0, PMID: 19866363

[15] Rubenstein JH Morgenstern H Longstreth K. Clustering of esophageal cancer among white men in the United States. Dis Esophagus. (2019) 32:doy081. doi: 10.1093/dote/doy081, PMID: 30169649 PMC6303731

[16] Gallaway MS Henley SJ Steele CB Momin B Thomas CC Jamal A. Surveillance for cancers associated with tobacco use - United States, 2010-2014. MMWR Surveill Summ. (2018) 67:1–42. doi: 10.15585/mmwr.ss6712a1, PMID: 30383737 PMC6220819

[17] Bhatia S Landier W Paskett ED Peters KB Merrill JK Phillips J. Rural-urban disparities in Cancer outcomes: opportunities for future research. J Natl Cancer Inst. (2022) 114:940–52. doi: 10.1093/jnci/djac030, PMID: 35148389 PMC9275775

[18] Wang X Allen MJ Espin-Garcia O Suzuiki C Bach Y Panov E. Outcomes in older adults with metastatic esophageal and gastric carcinoma treated with palliative chemotherapy. Oncologist. (2024) 29:e1501–10. doi: 10.1093/oncolo/oyae190, PMID: 39046894 PMC11546644

[19] Ghazwani Y Alghafees M Suheb MK Shafqat A Sabbah BN Arabi TZ. Trends in genitourinary cancer mortality in the United States: analysis of the CDC-WONDER database 1999-2020. Front Public Health. (2024) 12:1354663. doi: 10.3389/fpubh.2024.1354663, PMID: 38966707 PMC11223728

