# Supplementary figures and images for: Esophageal cancer mortality trends in the United States: a comprehensive longitudinal study (1999–2023) using CDC WONDER data
Source: Front Public Health. 2025 Oct 29;13:1696850. doi: 10.3389/fpubh.2025.1696850 (PMC12605218; doi:10.3389/fpubh.2025.1696850)

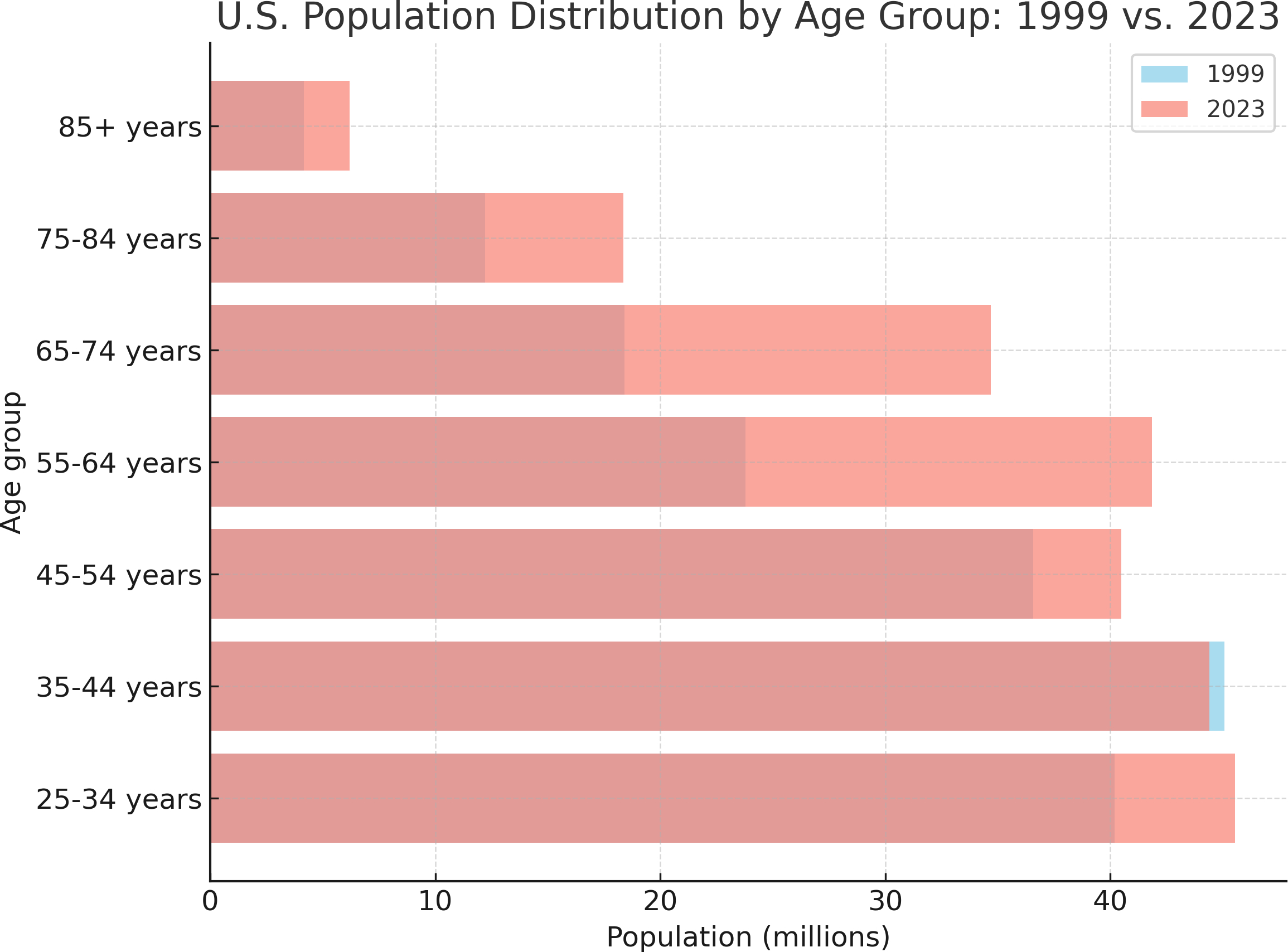

Supplement: SUPPLEMENTARY FIGURE S1 — Population age distribution of the United States in 1999 and 2023. Shown are the percentage shares by 5–year age groups for each year, normalized so that percentages sum to 100% within year. The figure highlights the shift toward older age groups over time. [file Image_1.tif]

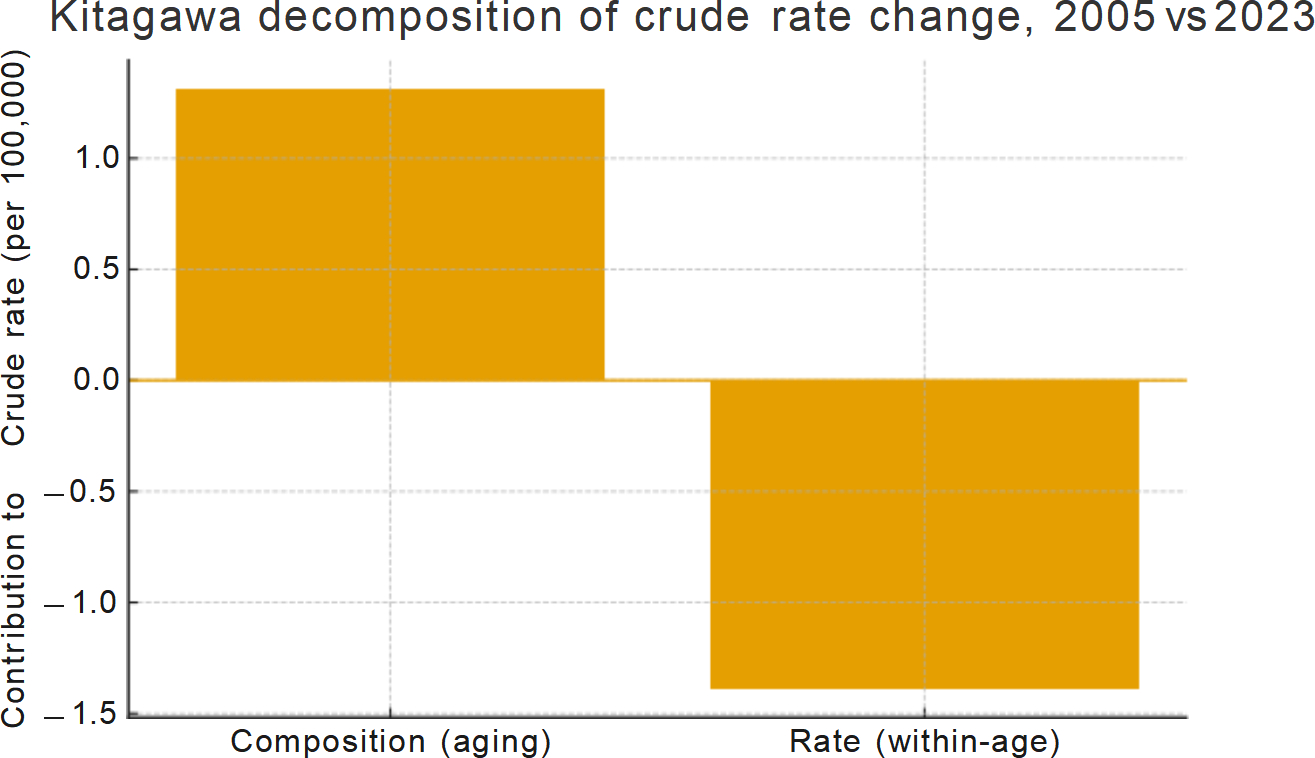

Supplement: SUPPLEMENTARY FIGURE S2 — Kitagawa decomposition of crude rate change, 2005–2023 (ages ≥25 years). Contributions (per 100,000) of population composition (age structure) versus within-age mortality changes to the net change in crude mortality rate between 2005 and 2023. Positive bars indicate an increase in the overall crude rate; negative bars indicate a decrease. [file Image_2.tif]

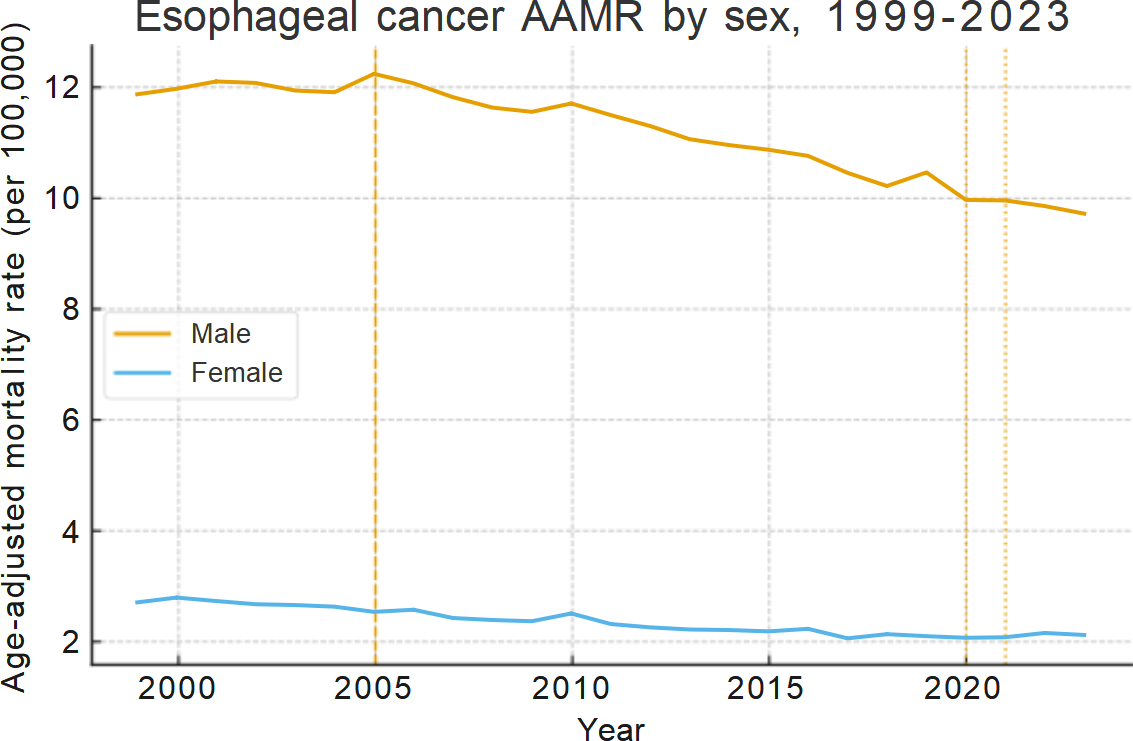

Supplement: SUPPLEMENTARY FIGURE S3 — Esophageal cancer AAMR by sex, 1999–2023.Age-adjusted mortality rates (per 100,000) for males and females, standardized to the 2000 U.S. standard population (5–year age groups). Vertical axis shows AAMR; horizontal axis shows calendar year. AAMR, age-adjusted mortality rate. [file Image_3.tif]

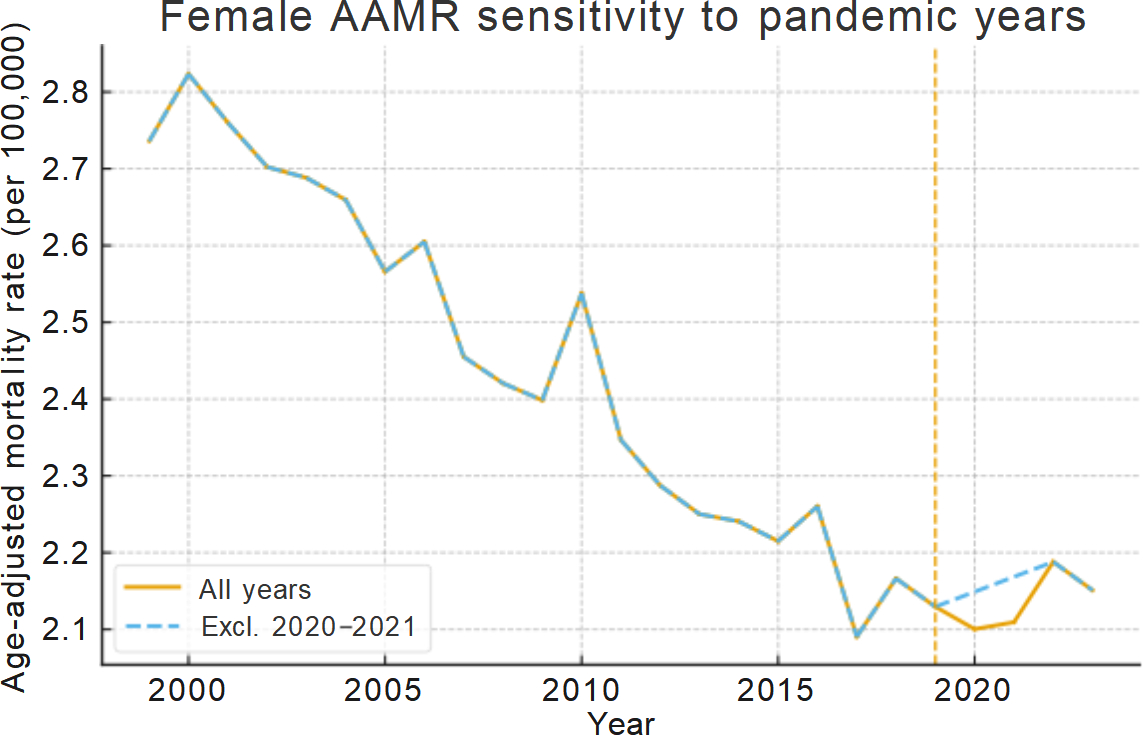

Supplement: SUPPLEMENTARY FIGURE S4 — Female AAMR sensitivity to pandemic years. Comparison of female AAMR trends using all years versus excluding 2020–2021. Rates are standardized to the 2000 U.S. standard population (5–year age groups). AAMR, age-adjusted mortality rate. [file Image_4.tif]

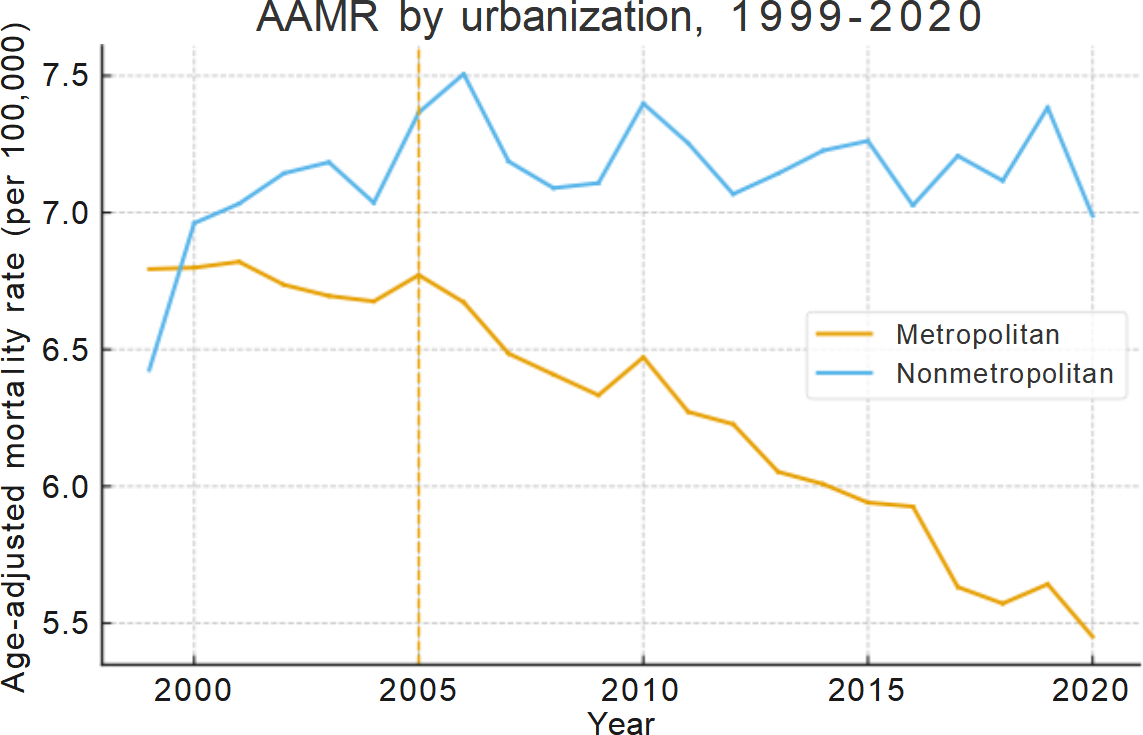

Supplement: SUPPLEMENTARY FIGURE S5 — AAMR by urbanization, 1999–2020. Age-adjusted mortality rates for metropolitan versus nonmetropolitan areas, using the fixed 2013 NCHS Urban Rural Classification (county assignments held constant). Rates are standardized to the 2000 U.S. standard population (5–year age groups). NCHS, National Center for Health Statistics. [file Image_5.tif]

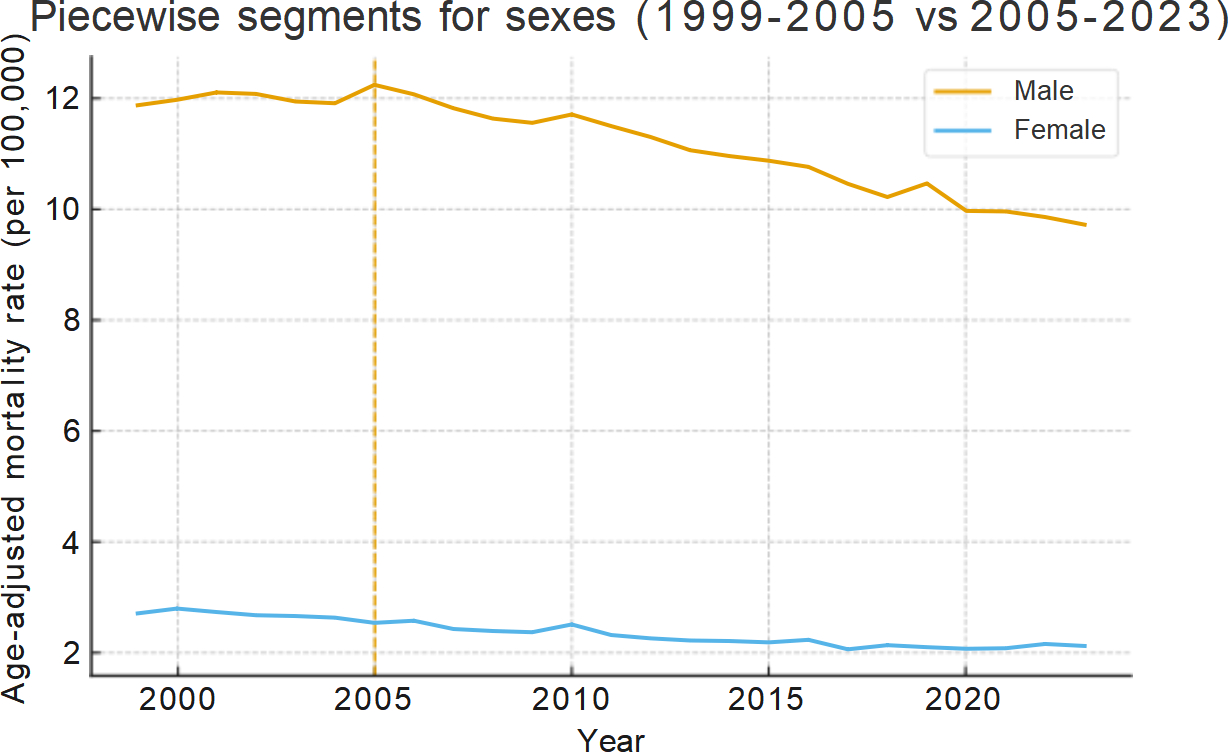

Supplement: SUPPLEMENTARY FIGURE S6 — Piecewise segments for sexes (1999–2005 vs 2005–2023). Sex-specific AAMR trends fit with simple log-linear models for the prespecified intervals 1999–2005 and 2005–2023. Rates standardized to the 2000 U.S. standard population (5–year age groups). [file Image_6.tif]
